# Epidemiology and survival outcome of breast cancer in a nationwide study

**DOI:** 10.18632/oncotarget.15207

**Published:** 2017-02-09

**Authors:** Fu-Chao Liu, Huan-Tang Lin, Chang-Fu Kuo, Lai-Chu See, Meng-Jiun Chiou, Huang-Ping Yu

**Affiliations:** ^1^ Department of Anesthesiology, Chang Gung Memorial Hospital, Taoyuan, Taiwan; ^2^ College of Medicine, Chang Gung University, Taoyuan, Taiwan; ^3^ Division of Rheumatology, Allergy and Immunology, Chang Gung Memorial Hospital, Taoyuan, Taiwan; ^4^ Division of Rheumatology, Orthopaedics and Dermatology, University of Nottingham, Nottingham, UK; ^5^ Department of Public Health, College of Medicine, Chang Gung University, T0aoyuan, Taiwan; ^6^ Biostatistics Core Laboratory, Molecular Medicine Research Center, Chang Gung University, Taoyuan, Taiwan; ^7^ Office for Big Data Research, Chang Gung Memorial Hospital, Taoyuan, Taiwan

**Keywords:** breast cancer, epidemiology, survival outcome, nationwide population study

## Abstract

Breast cancer is among the most prevalent cancers in Taiwan. The National Health Insurance database was used to identify patients with breast cancer and estimate the yearly prevalence and incidence of breast cancer between 1997 and 2013. Joinpoint regression analysis was used for the annual percentage change of incidence, prevalence, and survival outcome. Among 12,181,919 female beneficiaries in 2013, the prevalence was 834.37 per 100,000 persons (95% confidence interval, 829.28–839.45) and the incidence was 93.00 per 100,000 person-year (95% confidence interval, 91.27–94.73). The average annual percentage change of the age-standardized breast cancer incidence was 3.5 per 100,000 person-years (3.1–3.8; *P* < 0.05), suggesting an increase in breast cancer incidence over the study period. The 5-year mortality rate was 4.5% in 1997 and 4.4% in 2008. The 5-year mortality rate among patients with Charlson comorbidity index > 1 was 39.1% (19.2%–59.1%) in 1997 and 21.1% (15.7%-32.0%) in 2008, with an annual percentage change of –0.8 (–1.3 to 2.9), suggesting that the mortality rate was gradually decreasing in patients with comorbidities. In conclusion, 1 in 120 women in Taiwan has breast cancer and the incidence is rising, while the annual percentage change of breast cancer prevalence is decreasing. The mortality rate of breast cancer was essentially stable, but the 1-year, 2-year, and 5-year mortality rates in people with Charlson comorbidity index > 1 were declined.

## INTRODUCTION

According to GLOBOCAN 2012, breast cancer is the second most common cancer and the fifth most common cause of cancer deaths worldwide, with an age-standardized incidence rate (ASR) of 43.3 per 100,000 women-years and a worldwide mortality rate of 12.9% in 2012 [1]. The breast cancer incidence rate was considerably lower in Eastern Asia than in Northern America and Europe (ASR: 27.0, 91.6, and 71.1 per 100,000 women-years, respectively). Similarly, the breast cancer mortality rate was also lower (ASR: 6.2%, 14.8%, and 16.1%, respectively). In Asia, reports have indicated that the annual incidence of breast cancer has doubled or tripled over the past two decades. In Asia, the onset of breast cancer tends to occur at a younger age and more estrogen receptor (ER) positive or progesterone receptor (PR) positive subtypes, associated with a more favorable clinical-pathological outcome [2]. The etiology of breast cancer has generally been attributed to genetic, reproductive, and hormonal factors. In a breast cancer risk study conducted in Taiwan by Chuang et al. [3], estrogen-related factors, such as obesity, endometriosis, uterine myoma, hypertension, and dyslipidemia, were identified as important risk factors for patients with breast cancer. The known risk of female breast cancer includes *BRCA1* or *BRCA2* mutation, chest radiation exposure < 30 year, dense breast density, abnormal breast biopsy, family history of ovarian or breast cancer, late parity (age > 30 year) or nulliparity, early menarche (age < 12 year) or late menopause (age > 55 year), hormone-replacement therapy, postmenopausal obesity, white race, alcohol consumption and smoking [4]. In order to risk stratification of breast cancer for population-based screening, the Gail model was developed in 1989 to calculate an individual's combined risks of developing invasive breast cancer [5]. The Gail model was used widely since then but it is known to overestimate breast cancer risk for Asian women [5]. The updated Korean model proposed by Min et al. in 2014 [6], including risk factors of age, body mass index, menopause status, breast feeding, family history, previous breast test and age at first delivery, showed a better performance than the other models for Asian women.

Compared to female breast cancer, male breast cancer accounts for less than 1% of all breast cancers in the United States [7] and Korea [8]. Male breast cancer was diagnosed at early stages and high rate of ER positivity [8], but evidences revealed that aromatase inhibitors are less effective in males than in post-menopausal women [9]. Though most male breast cancer subtypes are invasive ductal carcinomas, the 5-year overall survival rate was comparable between male and female breast cancer patients in Korea [8] while poorer overall survival but better breast cancer-specific survival in Hong Kong [10]. In our NHI database, the incidence of male breast cancer was rare, accounted for 0.4% of all breast cancer (see Supplementary Table 1). Therefore, this study focused on epidemiology of female breast cancer in Taiwan.

Data regarding breast cancer epidemiology in Taiwan are rare, and surveys conducted by the International Agency for Research on Cancer (IARC) of World Health Organization (WHO) have not included Taiwan [1]. A recent study reported prevalence and incidence trends of multiple categories of cancer in Taiwan using the National Cancer Registry Database [11]. However, stratification of the estimates and mortality trends of breast cancer are lacking. This study aims to estimate the secular trends of the epidemiology of breast cancer, the association between breast cancer and preexisting comorbidities, and breast cancer mortality rates using the National Health Insurance (NHI) database.

## RESULTS

### Study population and socioeconomic status of patients with breast cancer

The study population comprised 22,080,199 registered NHI beneficiaries in Taiwan between 1997 and 2013. Of these, 125,253 female patients with breast cancer were identified. The average age of subjects was 52.57 ± 12.21 years. Patient baseline characteristics are presented in Table 1. Women living in rural areas comprised only one-third of breast cancer cases, while women in urban and suburban areas each represented one-third of cases. Furthermore, the prevalence of breast cancer was lower among women with lower income (quintile 1 and 2, total 30.83%) than among women with higher income (quintile 4 and 5, total 39.4%). The socioeconomic analysis indicated that breast cancer was more prevalent in high-income urban areas in Taiwan.

**Table 1:** Clinical characteristics of patients with breast cancer from 1997 to 2013

	Breast cancer cases(*n* = 125,253)	
**Age (years) (mean ± standard deviation)**	52.57 ± 12.21	
**Place of residence, No. (%)**		
** Urban**	41,710	(33.23%)
** Suburban**	37,159	(29.60%)
** Rural**	42,865	(34.15%)
** Unknown**	3,787	(3.02%)
**Income levels, No. (%)**		
** Quintile 1 (lowest)**	28,742	(22.90%)
** Quintile 2**	9,960	(7.93%)
** Quintile 3**	33,350	(26.57%)
** Quintile 4**	22,305	(17.77%)
** Quintile 5 (highest)**	27,149	(21.63%)
** Unknown**	4,015	(3.20%)
**Occupation, No. (%)**		
** Dependents of the insured individuals**	39,516	(31.48%)
** Civil servants, teachers, military personnel and veterans**	7,510	(5.98%)
** Non-manual workers and professionals**	21,936	(17.48%)
** Manual workers**	39,314	(31.32%)
** Other**	13,458	(10.72%)
** Unknown**	3,787	(3.02%)

### Prevalence and incidence of breast cancer between 1997 and 2013

**Figure 1:**
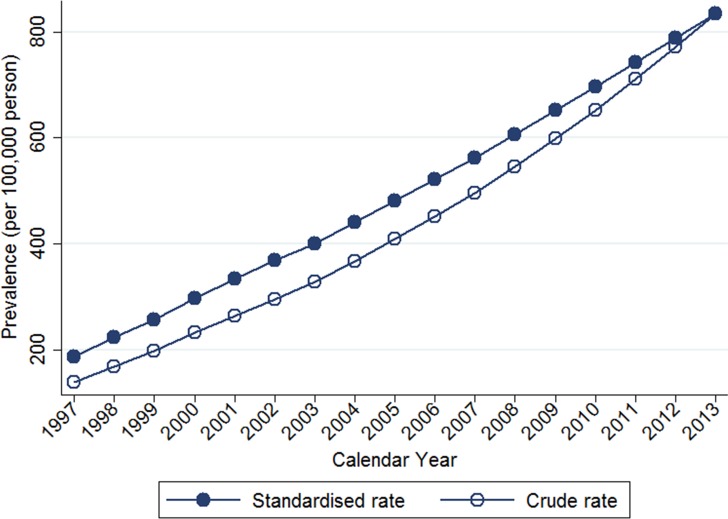
Trends of crude and age-standardized prevalence of breast cancer in Taiwan, 1997-2013

**Figure 2:**
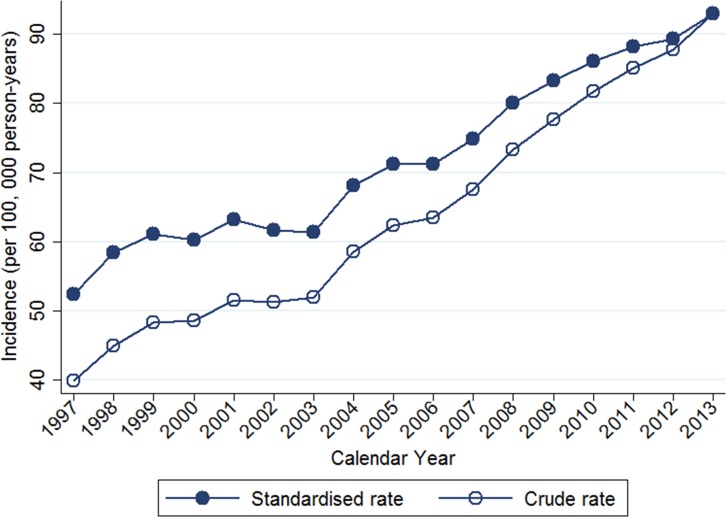
Trends of crude and age-standardized incidence of breast cancer in Taiwan, 1997-2013

**Figure 3:**
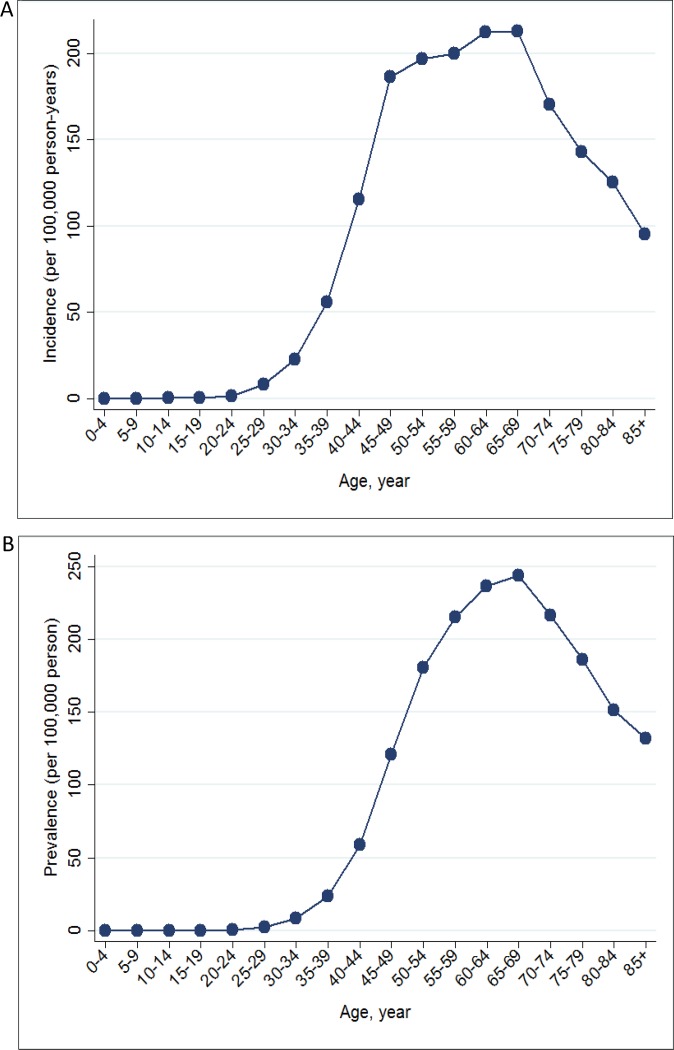
Age-specific prevalence (**A**) and incidence (**B**) of breast cancer in Taiwan in 2013.

Table 2 shows the temporal trends in breast cancer prevalence and incidence in Taiwan between 1997 and 2013. Overall, the age-adjusted standardized estimates were slightly higher than the crude estimates, namely due to the aging population effect over time. The standardized prevalence of breast cancer was 186.46 [95% confidence interval (CI): 183.38–189.53] per 100,000 person in 1997 and 834.37 (95% CI: 829.28–839.45) per 100,000 person in 2013 (Figure 1). The standardized incidence was 52.34 (95% CI: 50.70–53.98) per 100,000 person-year in 1997 and 93.00 (95% CI: 91.27–94.73) per 100,000 person-year in 2013 (Figure 2). Overall, the standardized prevalence and incidence of breast cancer were 4.5-fold and 1.8-fold higher, respectively, in 2013 than in 1997. The average APC of breast cancer incidence was 3.5 (95% CI: 3.1 to 3.8, *P* < 0.05), suggesting an increase in the incidence of breast cancer over the past two decades. Figure 3 shows the age-specific prevalence and incidence of breast cancer in 2013. The age-specific prevalence and incidence increased with age, with a peak at 65–69 years old, followed by a drop after the age of 70.

**Table 2:** Crude and age-standardized prevalence and incidence of breast cancer from 1997 to 2013

Year	Prevalence (per 100,000)					Incidence (per 100,000 person year)				
	N	Crude		Standardized		Person-years	Crude		Standardized	
**1997**	10438638	138.05	(135.80–140.31)	186.46	(183.38–189.53)	10107025.25	39.85	(38.62–41.08)	52.34	(50.70–53.98)
**1998**	10595158	167.43	(164.97–169.90)	222.51	(219.20–225.81)	10338003.88	44.90	(43.61–46.19)	58.39	(56.69–60.09)
**1999**	10740720	196.94	(194.29–199.60)	256.61	(253.12–260.09)	10480813.77	48.31	(46.98–49.64)	61.07	(59.37–62.77)
**2000**	10913556	231.84	(228.98–234.70)	296.99	(293.30–300.67)	10610031.40	48.60	(47.28–49.93)	60.28	(58.62–61.94)
**2001**	11144862	263.02	(260.01–266.03)	333.22	(329.38–337.05)	10756902.80	51.58	(50.22–52.93)	63.18	(61.51–64.86)
**2002**	11300005	295.86	(292.69–299.03)	368.97	(365.00–372.94)	10948634.14	51.28	(49.94–52.63)	61.69	(60.06–63.31)
**2003**	11408966	327.99	(324.66–331.31)	400.42	(396.35–404.48)	11125297.34	51.92	(50.58–53.26)	61.43	(59.84–63.03)
**2004**	11538714	367.25	(363.75–370.75)	439.53	(435.34–443.73)	11264172.68	58.59	(57.18–60.01)	68.13	(66.48–69.79)
**2005**	11651294	408.58	(404.91–412.25)	480.03	(475.71–484.35)	11384654.48	62.31	(60.86–63.76)	71.21	(69.55–72.88)
**2006**	11753283	451.17	(447.33–455.01)	520.88	(516.44–525.31)	11491272.21	63.44	(61.98–64.90)	71.17	(69.53–72.81)
**2007**	11835798	495.02	(491.01–499.02)	561.97	(557.42–566.52)	11584053.10	67.55	(66.05–69.05)	74.93	(73.26–76.60)
**2008**	11922720	544.67	(540.48–548.86)	606.85	(602.19–611.51)	11665456.60	73.39	(71.83–74.94)	80.01	(78.31–81.71)
**2009**	11985945	598.47	(594.09–602.85)	652.94	(648.18–657.70)	11736505.15	77.66	(76.07–79.26)	83.37	(81.66–85.09)
**2010**	12054764	651.87	(647.31–656.42)	696.21	(691.36–701.05)	11806110.41	81.76	(80.13–83.39)	86.15	(84.43–87.87)
**2011**	12115576	710.44	(705.69–715.19)	742.33	(737.41–747.26)	11856082.68	85.08	(83.42–86.74)	88.18	(86.46–89.90)
**2012**	12166485	770.99	(766.05–775.92)	788.41	(783.40–793.42)	11891015.64	87.75	(86.06–89.43)	89.36	(87.65–91.07)
**2013**	12181919	834.37	(829.28–839.45)	834.37	(829.28–839.45)	11870932.88	93.00	(91.27–94.73)	93.00	(91.27–94.73)

### Joinpoint regression analysis of breast cancer prevalence and incidence

Table 3A and 3B showed the joinpoint regression analysis of breast cancer incidence and prevalence, respectively. The average APC of breast cancer incidence was 3.5 (95% CI: 3.1 to 3.8, *P* < 0.05) per 100,000 person-years with zero joinpoint. The calculated best-fit joinpoint of breast cancer prevalence was 3, dividing the study period into 4 segments. The average APC of breast cancer prevalence was 17.1 (15.5–18.8) per 100,000 person-years, 1997–2000; 9.8 (9.2–10.5) per 100,000 person-years, 2000–2005; 7.9 (7.1–8.8) per 100,000 person-years, 2005–2009; and 6.3 (5.9–6.7) per 100,000 person-years, 2009–2013. The overall average APC was 9.8 (9.4–10.1) per 100,000 person-years between 1997 and 2013. This indicates gradual decrease in breast cancer prevalence over time.

**Table 3A:** Joinpoint analysis of breast cancer incidence in Taiwan, 1997-2013

Breast cancer incidence (per 100,000 person-years)					Trend	
**1997**		2013		Average APC	Years	APC (95%CI)
**52.34**	(50.70-53.98)	93.00	(91.27-94.73)	3.5 (3.1 to 3.8)	Zero joinpoint	

Abbreviations: APC, annual percent change; *P < 0.05

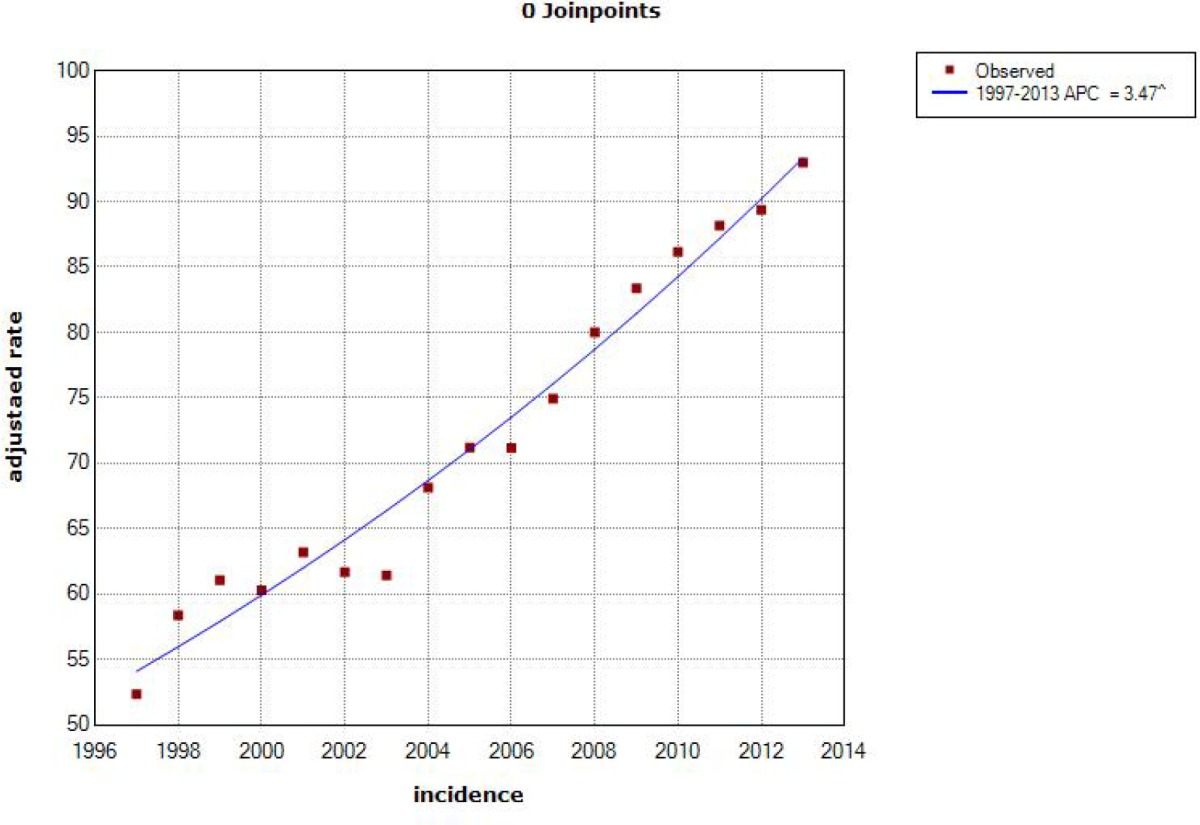

**Table 3B:** Joinpoint analysis of breast cancer prevalence in Taiwan, 1997-2013

Breast cancer prevalence (per 100,000 person)					Trend (3 joinpoint)							
1997		2013		Average APC	Segment 1	APC (95%CI)	Segment 2	APC (95%CI)	Segment 3	APC (95%CI)	Segment 4	APC (95%CI)
186.46	183.38–189.53	834.37	829.28–839.45	9.8*(9.4–10.1)	1997–2000	17.1* (15.5–18.8)	2000–2005	9.8* (9.2–10.5)	2005–2009	7.9* (7.1–8.8)	2009–2013	6.3* (5.9–6.7)

Abbreviations: APC, annual percent change; *P < 0.05

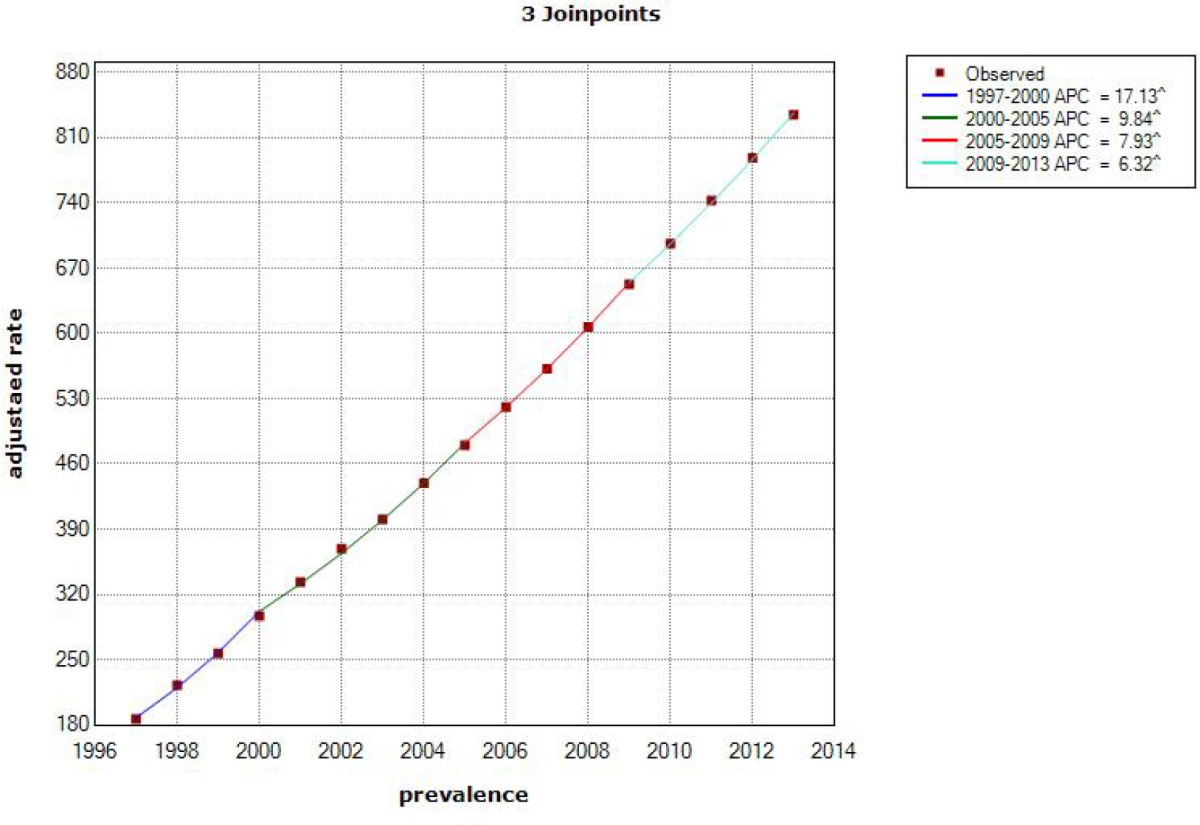

### Mortality rate of breast cancer

**Figure 4:**
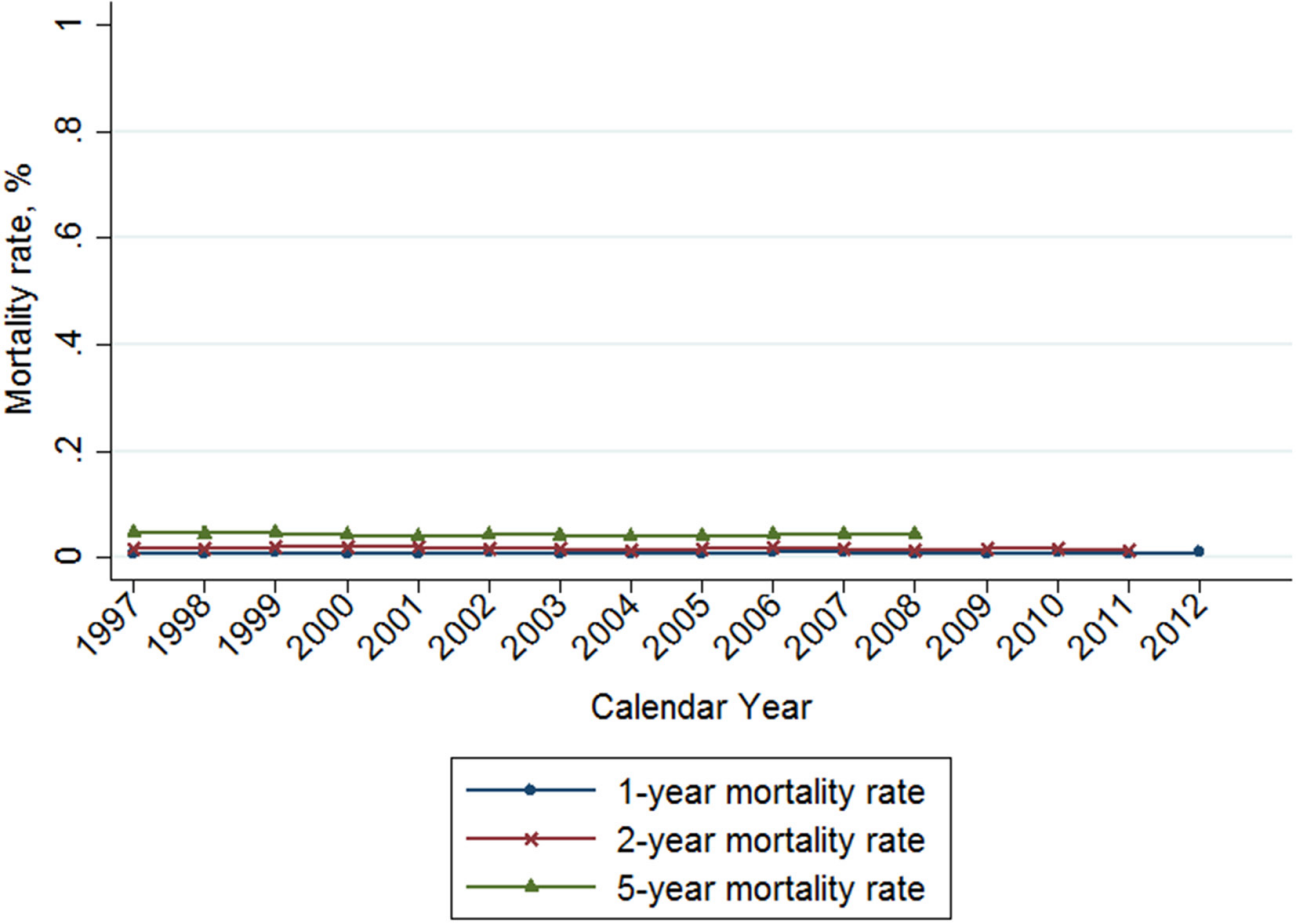
Trends of 1-, 2-, and 5-year mortality rate of breast cancer in Taiwan, 1997-2012

**Figure 5:**
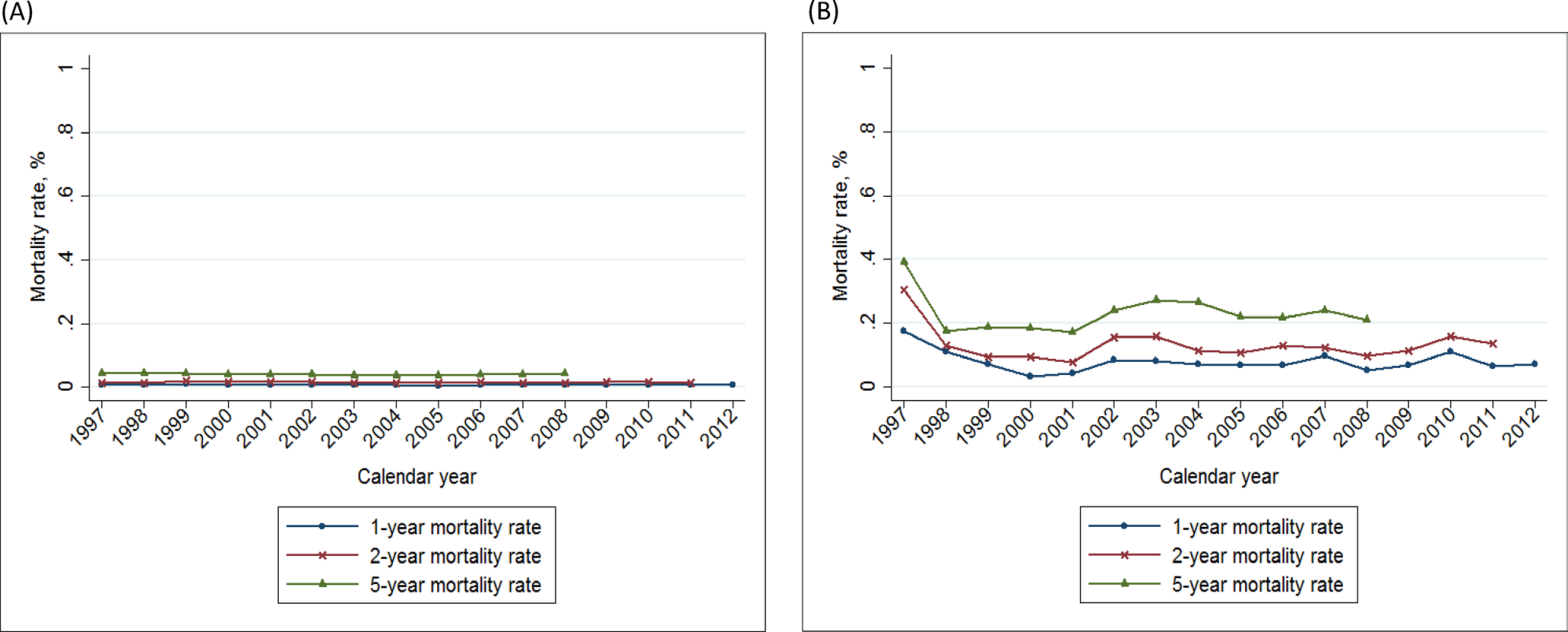
Trends of 1–, 2–, and 5– year mortality rate of breast cancer in Taiwan, 1997–2012, according to the Charlson comorbidity index (**A**): Charlson index ≤ 1; (**B**): Charlson index > 1 (yellow: 1-year mortality rate; green: 2-year mortality rate; purple: 5-year mortality rate).

The 1-year, 2-year and 5-year breast cancer mortality rates in Taiwan were stable (Figure 4), with zero average annual percent change during the study period (Table 4). The calculated mortality rates were as follows: the 1-year mortality rate was 0.9% in 2012, the 2-year mortality rate was 1.5% in 2011, and the 5-year mortality rate was 4.4% in 2008 (Table 4). In view of disease severity and patient comorbidity on survival outcome, we divided our study population into 2 groups, according to the Charlson comorbidity index (CCI). The CCI < 1 group included 98.7% of patients with breast cancer (Table 5) with a slightly reduced mortality rate compared to the general population (1-year: 0.8%, 2-year: 1.3%; 5-year: 4.2%). Conversely, the CCI > 1 group included 1.3% of patients with breast cancer with a much higher mortality rate (1-year: 7.1%, 2-year: 13.7%; 5-year: 21.1%). The average APC of 1-year, 2-year, 5-year mortality rates in the CCI > 1 group were -0.6 (–2.1 to 0.8), –0.3 (–0.2 to 0.7), and –0.8 (–1.3 to 2.9), respectively. There was an obvious decline in mortality rate in the CCI > 1 group during the study period (Figure 5).

**Table 4:** Joinpoint analysis of breast cancer mortality rate in Taiwan, 1997-2012

	Breast cancer survival rate				
	1997		Last year^a^		Average APC
Total					
1 year mortality rate	0.8	(0.5–1.0)	0.9	(0.7–1.1)	0.0 (0.0 to 0.0)
2 year mortality rate	1.5	(1.2–1.9)	1.5	(1.2–1.7)	0.0 (0.0 to 0.0)
5 year mortality rate	4.5	(3.8–5.1)	4.4	(3.8–4.7)	0.0 (–0.1 to 0.0)
CCI					
CCI ≤ 1					
1 year mortality rate	0.7	(0.4–0.9)	0.8	(0.6–1.0)	0.0 (0.0 to 0.0)
2 year mortality rate	1.4	(1.0–1.7)	1.3	(1.1–1.6)	0.0 (0.0 to 0.0)
5 year mortality rate	4.3	(3.6–4.9)	4.2	(3.5–4.4)	0.0 (–0.1 to 0.1)
CCI > 1					
1 year mortality rate	17.4	(1.9–32.9)	7.1	(2.4–11.9)	–0.6 (–2.1 to 0.8)
2 year mortality rate	30.4	(11.6–49.2)	13.7	(7.7–19.8)	–0.3 (–0.2 to 0.7)
5 year mortality rate	39.1	(19.2–59.1)	21.1	(15.7–32.0)	–0.8 (–1.3 to 2.9)

Abbreviations: APC, annual percent change; *P < 0.05.

^a^Last year: 1-year mortality rate for 2012; 2-year mortality rate for 2011; 5-year mortality rate for 2008.

**Table 5:** Number of female patients with breast cancer grouped by the Charlson comorbidity index

Year	Charlson comorbidity index		
	< 1	> 1	Total
1997	4005	23	4028
	99.43	0.57	
1998	4596	46	4642
	99.01	0.99	
1999	4978	85	5063
	98.32	1.68	
2000	5059	98	5157
	98.1	1.9	
2001	5432	116	5548
	97.91	2.09	
2002	5519	96	5615
	98.29	1.71	
2003	5688	88	5776
	98.48	1.52	
2004	6502	98	6600
	98.52	1.48	
2005	6990	104	7094
	98.53	1.47	
2006	7188	102	7290
	98.6	1.4	
2007	7720	105	7825
	98.66	1.34	
2008	8447	114	8561
	98.67	1.33	
2009	8999	116	9115
	98.73	1.27	
2010	9533	120	9653
	98.76	1.24	
2011	9963	124	10087
	98.77	1.23	
2012	10322	112	10434
	98.93	1.07	
2013	10949	91	11040
	99.18	0.82	
Total	121890(98.67%)	1638(1.3%)	123528

## DISCUSSION

Four main intrinsic molecular subtypes of breast cancer have been identified based on the expression of ER, PR, human epidermal receptor-2 (HER2) [12, 13]. The loss of hormone receptor positivity and the switch to the triple negative phenotype after neoadjuvant therapy were associated with a worse patient outcome [14, 15]. The heritability of breast cancer has been estimated to be about 31% [16]. Known genetic factors contributing to a higher lifetime risk of breast cancer comprise rare variants in *BRCA1, BRCA2, PALB2, ATM* and *CHEK2* [17]. Taken together, these genetic variants explain about 37% of the excess familial breast cancer risk [17]. Recent advances in sequencing technology have made panel multigene testing practical for risk stratification of invasive breast cancer according to genetic variants [18]. The clinical utility of several genomic tests such as 21-gene expression assay [19] or 70-gene signature [20] have been proved in prospective long-term clinical trials to better predict clinical outcomes.

The prevalence and incidence of breast cancer have been rising globally [21]. However, breast cancer mortality rates have been stable, or have slightly declined. In the USA, breast cancer mortality rates decreased by 36% between 1989 and 2012 [22]. Sung et al. [23] found comparable longitudinal breast cancer age-specific incidence rates (ASR) among Asian and Western populations. Taiwan implemented a stratified breast cancer screening program in 1995 [24]. Since 2002, the Taiwan government has provided a national biennial mammography screening program for women aged between 40 and 69 years [24]. According to the Ministry of Health and Welfare in Taiwan, 690,000 women underwent screening mammography in 2013, with a screening rate of 36% [11]. Amy Ming-Fang Yen et al. [25] investigated screening efficiency in Taiwan and found that population-based screening mammography was associated with a 41% reduction in breast cancer mortality rates.

Our study indicated that there was a rising trend in the prevalence and incidence of breast cancer in Taiwan, while mortality rates were essentially stable. These findings were consistent with epidemiological studies conducted in other countries [21, 26]. A study involving adult cancer patients, based on the Taiwan cancer registries database [11], found that between 2002 and 2012 the breast cancer incidence APCs in men and women were 4.9%, and -2.4% respectively, and the 5-year age-standardized relative survival rate was 82.8% in 2008. These findings are consistent with our results.

In countries with a universal health care system, higher socioeconomic status is associated with better overall survival, while lower socioeconomic status is correlated with increased cancer mortality [27–29]. Our socioeconomic status analysis showed a similar pattern, with a reduced prevalence of breast cancer cases in low-income rural areas. Furthermore, emerging treatment modalities have contributed to a reduction in breast cancer mortality rates, particularly in patients with multiple comorbidities. These advances may explain the significant improvement in the survival rate of the CCI > 1 group in our study.

Standardized clinical approaches to breast cancer include breast imaging, surgery, pathological analysis and subtyping, radiotherapy, and near universal application of adjuvant systemic therapy [30]. Systemic treatments have proved to be effective at reducing distant metastasis and local recurrence rate to less than 5% during 10 years of follow-up after breast surgery [31, 32]. For example, adjuvant therapy with pan-HER2 tyrosine-kinase inhibitor neratinib significantly improved invasive disease-free survival in women after chemotherapy and trastuzumab-based adjuvant therapy with HER2-positive early-stage breast cancer [33]. Recent large-scale cohort studies revealed that screening mammography resulted in reduction of breast cancer-specific mortality [34, 35].

There are several limitations to our study. The NHI released de-identified and encrypted data for public research, so there are no clues of clinical information on cancer staging or treatment modalities, which could only be obtained by the Taiwan Cancer Registry Database. However, we validated breast cancer diagnosis against the National Cancer Registry, a highly accurate source for the diagnosis of cancer. The agreement between the NHI database and the National Cancer Registry breast cancer diagnoses is excellent. Furthermore, NHI database has a 99.9% coverage of the population in Taiwan while the Cancer Registry Database had a 98.4% coverage of the population in 2012. These data are inherently complementary and are supported by consistent epidemiologic statistics between previous studies [11] and our results. Finally, the prevalence reported in this study was defined within a 10-year period, rather than a lifetime prevalence, which could be higher theoretically. Overall, we may have underestimated breast cancer prevalence rates but overestimated incidence rates.

In conclusion, this epidemiological study of breast cancer in Taiwan found a rising trend in breast cancer prevalence and incidence. However, the AAPC of breast cancer prevalence by joinpoint regression analysis showed a gradual deceleration of the increasing trend. The breast cancer mortality rate was essentially stable during the study period, but we observed a reduction in the 1-year, 2-year, and 5-year mortality rates in patients with CCI > 1. By improving patient awareness, and providing efficient screening and innovative treatments, we may be able to control the rising incidence of breast cancer and reduce mortality rates in Taiwan in the near future.

## MATERIALS AND METHODS

This study was approved by the Institutional Review Board of the Chang Gung Memorial Hospital (approval number 104-6697B) and the National Health Research Institute, the data holder of the National Health Insurance (NHI) research database.

### Data sources and study population

Our primary data source was the NHI research database. This database routinely collected health data for all individuals eligible for NHI. By law, all citizens are required to enroll in the program, resulting in an exceptionally high population coverage rate of over 99%. Our study database comprised approximately 28 million (living and deceased) beneficiaries, registered between 1 Jan 1997 and 31 Dec 2013. The NHI diagnostic coding system follows the International Classification of Diseases, Ninth Revision, Clinical Modification (ICD-9-CM). The reliability, representativeness, and clinical consistency of this database have been reported previously. In Taiwan, patients with major illnesses, such as cancer, are entitled to a medical copayment waiver. A diagnosis from a specialist and a review by a commissioned expert panel are required for approval of the waiver. The registry of patients with catastrophic illness records the clinical and administrative information of patients receiving this waiver. We identified patients with breast cancer through the registry using an ICD-9 CM code of 174.9. Denominator data were based on the Registry of Beneficiaries, containing the demographics, insurance status, residence, and socioeconomic data.

To ascertain the validity of breast cancer diagnosis in the NHI database, we used the National Cancer Registry as a standard to estimate the positive predictive value (PPV) and the negative predictive value (NPV) of breast cancer diagnosis. We also compared the databases for the estimated mortality rates. The PPV, NPV, sensitivity, and specificity of breast cancer diagnosis were 0.92, 1.00, 0.97, and 1.00, respectively.

### Estimation of prevalence and incidence

Prevalent cases of breast cancer were defined as individuals with at least one primary diagnosis of breast cancer within the 10-year period before 1 January of each calendar year. Prevalence was calculated by dividing the number of prevalent cases of breast cancer by the eligible population in a specified calendar year. Incident breast cancer cases were defined as beneficiaries with a registration period of at least 1 year prior to 1 January of each calendar year. For the incidence of breast cancer, we constructed at-risk cohorts for each calendar year, comprising all beneficiaries registered during the given calendar year without a history of breast cancer before 1 January of that year. The incidence was calculated using the number of incident breast cancer cases during a calendar year as the numerator and the total person-years in the at-risk population accumulating during that same year as the denominator.

### Trends of prevalence, incidence of breast cancer

To determine the trends in prevalence and incidence of breast cancer, we calculated age standardized prevalence and incidence of breast cancer in each calendar year between 1997 and 2013, with the population structure in 2013 as the reference. Joinpoint regression analysis was used to compare the average annual percent change (APC) of breast cancer prevalence and incidence during the study period.

### Trends of mortality rate of breast cancer

To determine the mortality rate of breast cancer, we constructed cohorts for each calendar year. The 1-, 2- and 5- year mortality rates of breast cancer were calculated during the specified calendar year using deceased breast cancer beneficiaries diagnosed after 1, 2 or 5 years of that year. We then divided our study population according to the Charlson comorbidity index (CCI) into 2 groups, CCI > 1 and CCI < 1, in order to compare the trend of 1–, 2–, 5– year mortality rate between 1997 and 2013.

### Statistical analysis

The 95% confidence intervals (CIs) of prevalence and incidence were based on the assumption of Poisson distribution for the observed prevalent and incident cases. We used the Joinpoint Regression Program (version 4.0.4; National Cancer Institute, Bethesda, MD, USA) to estimate trends in the prevalence and incidence of breast cancer. The Bayesian information criterion was used to generate different ‘joinpoints’ when the linear trend of prevalence and incidence of breast cancer changed significantly and to determine the best-fit situations. Average annual percentage changes (AAPCs) for each segment were calculated. The significance level was set at 0.05. All statistical analyses were conducted on SAS statistical software, version 9.3 (SAS Institute, Cary, NC, USA).

## SUPPLEMENTARY MATERIALS FIGURES AND TABLES



## Abbreviations

(NHI): National Health Insurance
(CI): confidence interval
(APC): annual percentage change
(CCI): Charlson comorbidity index
(ASR): age-standardized incidence rate
(ER): estrogen receptor positive
(PR): progesterone receptor
(IARC): International Agency for Research on Cancer
(WHO): World Health Organization
(ICD-9-CM): International Classification of Diseases
(HER): Ninth Revision
(SEER): Clinical Modification, human epidermal receptor, the Surveillance, Epidemiology, and End Results.
