# Supplementary material for: Epidemiology and survival outcome of breast cancer in a nationwide study
Source: Oncotarget. 2017 Feb 9;8(10):16939–50. doi: 10.18632/oncotarget.15207 (PMC5370012; doi:10.18632/oncotarget.15207)
Supplement: Supplementary file 1 [file oncotarget-08-16939-s001.pdf]

# Epidemiology and survival outcome of breast cancer in a nationwide study

## Supplementary Materials

**Supplementary Table 1: Cumulative frequency of male breast cancer in Taiwan**

| Year | Frequency | Percent | Cumulative Frequency |
|---|---|---|---|
| **1997** | 10 | 1.63% | 10 |
| **1998** | 27 | 4.40% | 37 |
| **1999** | 21 | 3.43% | 58 |
| **2000** | 28 | 4.57% | 86 |
| **2001** | 30 | 4.89% | 116 |
| **2002** | 23 | 3.75% | 139 |
| **2003** | 25 | 4.08% | 164 |
| **2004** | 61 | 9.95% | 225 |
| **2005** | 54 | 8.81% | 279 |
| **2006** | 52 | 8.48% | 331 |
| **2007** | 33 | 5.38% | 364 |
| **2008** | 41 | 6.69% | 405 |
| **2009** | 45 | 7.34% | 450 |
| **2010** | 45 | 7.34% | 495 |
| **2011** | 48 | 7.83% | 543 |
| **2012** | 28 | 4.57% | 571 |
| **2013** | 42 | 6.85% | 613 |
